# Current Approach in the Management of Secondary Immunodeficiency in Patients with Hematological Malignancies: Spanish Expert Consensus Recommendations

**DOI:** 10.3390/jcm12196356

**Published:** 2023-10-04

**Authors:** Concepción Boqué, Silvia Sánchez-Ramón, Raúl Córdoba, Carol Moreno, Elena Cabezudo

**Affiliations:** 1 Department of Hematology, Hospital Duran i Reynals, Institut Català d’Oncologia, 08908 Hospitalet de Llobregat, Barcelona, Spain; 2 Department of Clinical Immunology, Instituto de Medicina de Laboratorio, Hospital Clínico San Carlos, Universidad Complutense, 28040 Madrid, Spain; ssramon@salud.madrid.org; 3 Department of Hematology, Fundación Jimenez Diaz University Hospital, Health Research Institute-Fundación Jimenez Diaz, 28040 Madrid, Spain; raul.cordoba@fjd.es; 4 Department of Hematology, Hospital de la Santa Creu i Sant Pau, Josep Carreras Leukaemia Research Institute, Universitat Autónoma de Barcelona, 08025 Barcelona, Catalonia, Spain; cmoreno@santpau.cat; 5 Service of Hematology, ICO-Hospital de Sant Joan Despí Moisès Broggi, 08970 Sant Joan Despí, Barcelona, Spain; ecabezudoperez@gmail.com

**Keywords:** secondary immunodeficiency, B-cell lymphoproliferative disorders (BCLPD), infections, hypogammaglobulinemia, intravenous IgG

## Abstract

A Delphi-based survey was designed to assess the opinions of clinical hematologists (n = 17) and clinical immunologists (n = 18) from across Spain on secondary immunodeficiencies (SID) in the management of oncohematological patients. There was 100% agreement on the need to have available guidelines for the management of immunodeficiency in hematological patients; to perform a baseline immunological evaluation in patients with chronic lymphocytic leukemia (CLL), multiple myeloma (MM), lymphoma and hematopoietic stem cell transplantation (HSCT) recipients; and to quantify serum IgG, IgA and IgM levels when SID is suspected. More than 90% agreed on the need for active immunization against seasonal influenza and H1N1, pneumococcus and *Haemophilus influenzae*. There was a consensus on the monitoring of IgG levels every 3 months (83%) and the need to have available a clinical protocol for the use of IVIG in the management of SID (94%), to monitor trough IgG levels to determine the correct IVIG dose (86%) and to discontinue IVIG after the recovery of IgG levels after 12 months of follow-up (77%). The findings of the present survey may be useful recommendations for hematologists and immunologists to improve the management of SID in daily practice.

## 1. Introduction

Primary immunodeficiency diseases are genetic disorders that result in the partial or full impairment of the immune system components, leaving the patient unable to effectively resolve infections or displaying dysregulation. Secondary immunodeficiency diseases (SID) are the result of a disease or other environmental factors weakening the immune system. Patients with hematological diseases often present SID either because of adaptive or innate defects in the immune response related to the direct effects of malignant cells or to the effects of pharmacological treatments used in the management of hematological disorders or neoplasms, including hematopoietic progenitor transplants and graft vs. host disease (GvHD) [1,2].

The most frequent SID is seen in chronic lymphocytic leukemia (CLL), a B-cell lymphoproliferative disorder (BCLPD) with an altered immune response, such as antibody production, resulting in inadequate immunoglobulin (Ig) concentrations and the malfunctioning of polyclonal Ig, besides anomalous activity of the neoplastic and non-neoplastic B cells [2]. In fact, infections are the main cause of morbidity and mortality in CLL [3,4]. Correspondingly, patients with CLL and normal IgG levels may have a reduced response to immunization (e.g., pneumococcus vaccine), suggesting that the total IgG concentration alone is not enough to identify those patients who are at increased risk of infection [5]. Treatments can also have a deleterious effect on the immune systems of patients with CLL [3].

Multiple myeloma (MM) is generally recognized as another BCLPD, associated with intense immunodeficiency [6], being infections the cause of fatal outcomes in one out of five subjects with MM. The mechanisms by which MM patients develop SID include the involvement of innate and adaptive effectors of the immune system, such as immunoglobulin deficiency, anomalous immunoglobulin synthesis and cell-mediated immunity and natural killer (NK) cell defects, among others. The treatment of MM has also a great influence on SID because it is usually based on high doses of steroids, besides different strategies and new drugs with immunosuppressive effects, like B-cell-depleting therapies. The presence of secondary hypogammaglobulinemia, defined as IgG levels < 5 g/L, has been identified as an independent prognostic factor of survival in MM patients [7].

In patients with non-Hodgkin lymphoma (NHL) and Hodgkin disease (HL), also a BCLPD, SID can be frequently attributed to treatment including stem cell transplantation. Meanwhile, in hematopoietic transplant patients, SID is initially related to chemotherapy and immunosuppressive regimens to prevent GvHD, and subsequently to immunological alterations established between tolerance and surveillance after transplantation [2].

There is evidence of the beneficial effect of immunoglobulin replacement therapy (IgRT) in patients with secondary antibody deficiency (SAD) [6,7,8,9,10,11,12]. SAD is the most common type of SID, defined as a quantitative or qualitative decrease in antibodies that occurs most commonly as a consequence of hematological malignancies (mainly BCLPD), renal or gastrointestinal immunoglobulin loss and corticosteroid or other immunosuppressive or anticonvulsant medications. SAD can have a clinical course with limited infection susceptibility to more significant or life-threatening infectious episodes. Regarding the prescription of IgRT, the recommendations of the European Medicines Agency (EMA), effective as of January 1, 2019, include the indication of IgRT in all SID patients with severe or recurrent infections, ineffective antimicrobial treatment and either proven specific antibody failure to pneumococcal polysaccharide and polypeptide antigen vaccines (failure to mount at least a two-fold rise in IgG antibody titer) or a serum IgG level of <4 g/L (hypogammaglobulinemia) [13,14].

The incidence of SID is increasing due to the rapid expansion of the therapeutic armamentarium for lymphoproliferative disorders (LPD) in hematological malignancies. This growth is facilitated by the use of hematopoietic transplantation, chemotherapy, more specific immunosuppressive therapy and new biological agents that target B cells beyond rituximab. These include other B-cell-directed therapies (plasma cells) such as daratumumab (a recombinant monoclonal antibody that targets CD38 and depletes plasma cells), or those that target B cells, such as T-cell therapy with a chimeric antigen receptor (CAR), like anti-CD19 CAR-T-cell therapy or B-cell maturation antigen (BCMA)-directed CAR-T-cell therapy [1,15,16,17,18,19,20,21,22]. More recently, other novel biological agents have emerged that target T cells, including bispecific T-cell engagers (BiTEs).

Although there are clinical guidelines and recommendations on the use of IgRT in patients with CLL or treatment with anti-CD19-CD20 agents [23], in clinical practice, there is a lack of protocols about laboratory assessment and management, as well as accurate biological factors that could help clinicians to define SID. Basic screening procedures include, in general, a complete cell blood count, quantification of serum Ig and a biochemical panel of lymphocyte subpopulations. However, other tests that can be useful, such as antibody production assessment after vaccination or IgG subclasses, are still not considered essential work-up tests. In conclusion, there are no standardized protocols for the assessment and management of patients with BCLPD and SID.

The present questionnaire survey using the Delphi method was designed and conducted with the participation of a panel of expert hematologists and immunologists between December 2018 and March 2020, with two objectives: (a) to provide data on the real-world clinical practice in patients with SID and BCLPD and (b) to develop consensus recommendations for the management of SID in hematological malignancy patients, including different aspects of treatment with IgRT, as well as to assess points of agreement and disagreement between specialists in hematology and immunology. It should be noted that this questionnaire, primarily focused on baseline immunological assessments in patients with B-cell neoplasms, did not address post-hematopoietic stem cell transplant (HSCT) immunizations. The consensus statements provide a “first glimpse” into the clinical practices in Spain concerning the management of SID in hematological malignancies.

## 2. Materials and Methods

### 2.1. Survey Design

A questionnaire, named “IDSTATUS: Present and Future of the Management of Hematological Patients” (IDSTATUS is the Spanish acronym for Secondary Immunodeficiency Status), was designed with the support of the Spanish Society of Hematology and Hemotherapy (SEHH) and the Spanish Society of Immunology (SEI). The objectives of the survey were the following: (1) to determine the opinions and details of the routine clinical practice of experts in hematology and immunology in the management of patients with hematological malignancies and SID and (2) to develop a consensus among participants in order to optimize the care of these patients.

A modified Delphi method was used to reach a consensus. The original Delphi method involves three or more rounds, whereas the modified technique is limited to one or two rounds to avoid a decrease in the rate of acceptable responses due to the prolonged duration of the process [24].

### 2.2. Survey Questionnaire and Participants

At the beginning of the project, a scientific committee formed by five specialists (four hematologists and one immunologist) with proven experience and interest in SID in hematological malignancies was established. The scientific committee was responsible for the development of the Delphi questionnaire, and one hematologist and one immunologist coordinated the survey. The design of the questionnaire included three phases: (1) an initial proposal of the scope of the questionnaire by the Delphi coordinators after an extensive review of the literature and drafting of the different items, (2) a meeting of the members of the expert committee to discuss and modify the content of the questionnaire and (3) the final process of the development and validation of the definitive questionnaire by the expert committee.

The questions were grouped into two sections (A and B) and addressed to participants who were hematologists and immunologists, respectively. The questions included in section A were limited to data related to the current clinical practice in the management of SID in patients with hematological malignancies, grouped as CLL, MM, lymphoma, HSCT and advanced age/fragile patients. Questions included in section B were related to recommendations for the management of these patients. Each section (A and B) was divided into the following four subsections: (1) baseline immunological evaluation, (2) prophylaxis of infection, (3) treatment with intravenous IgG (IVIG) and (4) follow-up and monitoring of patients receiving IVIG. Section A included an additional subsection of “others” that included questions on treatment strategies and the frequency of infections related to the management of SID in hematological patients in routine daily practice. Moreover, the questionnaire included a preliminary part to gather demographic data and the characteristics of participants.

A newsletter with details of the objectives and characteristics of the IDSTATUS project was mailed to hematologists included in the database of the SEHH and also to the Spanish Group of Geriatric Hematology (GEHG) and to immunologists included in the database of the Spanish Society of Immunology (SEI). The Delphi questionnaire was lodged in an Internet microsite that participants accessed via a weblink, and those who agreed to take part in the survey were provided with the microsite URL and the user’s password. Participation in the survey was anonymous, voluntary and unpaid.

### 2.3. Assessment

Only fully completed questionnaires were considered. Each question in section A was formulated so that it could be answered using a 4-point Likert scale, including 1 = “never”, 2 = “sometimes”, 3 = “frequently” and 4 = “always”, according to the participant’s opinion regarding current clinical practice, whereas each question in section B, also using a 4-point Likert scale, could be answered as 1 = “not necessary, 2 = “optional, 3 = “recommendable” and 4 = “indispensable” regarding recommendations for the management of SID in hematological patients. Questions in the subsection of “others” could be answered as 1 = “strongly disagree”, 2 = “disagree”, 3 = “agree” and 4 = “strongly agree”.

A consensus was established in favor of the question/recommendation when the sum of the responses “frequently”, “recommendable” or “agree” (Likert score 3) and “always”, “indispensable” or “strongly agree” (Likert score 4) was equal to or greater than two thirds (66.6%) of the total responses obtained for that item. By contrast, a consensus against the question/recommendation was reached when the sum of responses “sometimes”, “optional” or “disagree” (Likert score 2) and “never”, “not necessary” or “strongly disagree” (Likert score 1) was equal to or greater than 66.6% of the total responses obtained for that item. When none of these previous assumptions were met, a consensus neither in favor nor against the statement was not reached.

**Figure 1:**
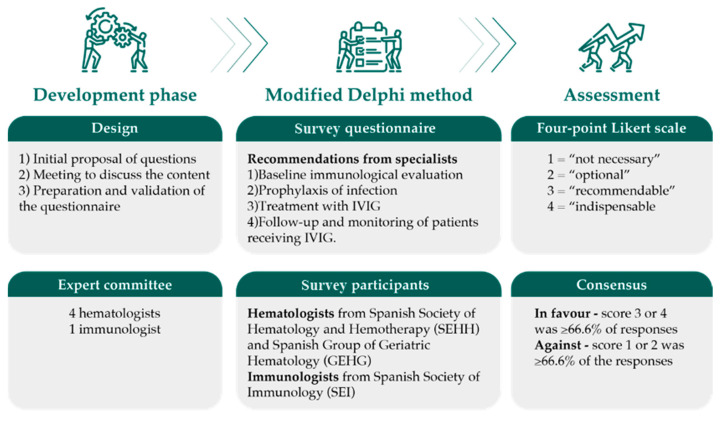
Design of the survey using the modified Delphi-based method.

A diagram of the methodology is shown in Figure 1.

## 3. Results

### 3.1. General Characteristics of Participants

Of a total of 88 questionnaires, 35 (39.8%) fully completed questionnaires were analyzed. The remaining 53 questionnaires, 24 from the hematologists and 29 from the immunologists, were returned incomplete and were excluded from the survey. The general characteristics of participants (hematologists, n = 17, immunologists = 18) are shown in Table 1.

Overall, 54% of participants reported more than 15 years of professional experience and 29% had between 5 and 10 years. The model of the hospital where the 94% of the participants developed their activity was a public center and 83% of them had more than 400 beds. In 65% of the centers, there was a clinical immunology consultation and in 75% there was a laboratory of immunology. All participants attended patients with SID due to hematological malignancies, with >30 patients/year in 41% of cases, 20–30 patients/year in 18%, 10–20% patients/year in 32% and 0–10 patients/year in 9%. However, only 35% of participants reported that they knew whether a protocol for the management of SID in oncohematological patients was available in their centers.

### 3.2. Current Clinical Practice

#### 3.2.1. Baseline Immunological Assessment

In the specific question regarding the performance of a baseline immunological evaluation in the initial survey of different conditions, which was addressed to clinical hematologists only, a consensus was obtained in four of the five items (Table 2).

In the remaining part of the questionnaire, a consensus was obtained by both hematologists and immunologists in most items, including the performance of a baseline immunological evaluation in the participant’s own center (88.6%), the need for a detailed medical history (85.7%) and physical examination (88.6%), the biochemical analysis of total proteins and protein electrophoresis (100%) and the quantification of IgG, IgA and IgM levels (97.1%). In the case of suspicion of SID, clinical immunologists achieved a consensus on the need for the assessment of IgG subclasses (72.2%), IgG antibody titers to previous immunization/exposures (77.8%) and specific antibodies against immunizations with protein and polysaccharide antigens (tetanus toxoid, *Salmonella typhi*, etc.) (66.7%), while hematologists did not reach a consensus regarding these items (23.5%). The immunophenotyping of subpopulations of T, B and NK cells was considered necessary by clinical immunologists only (83.3%). A consensus in favor of memory B-cell immunophenotyping was not reached. Detailed responses to all items included in this part of the questionnaire are shown in the Supplementary Materials Table S1.

#### 3.2.2. Prophylaxis of Infection

In this part of the survey, an overall consensus in favor of active immunization against seasonal influenza/H1N1 and pneumococcus infection for patients with CLL, MM and lymphoma was obtained, with overall percentages of agreement ranging between 71.4% and 97.1% (Table 3). Regarding the use of antibiotic prophylaxis for recurrent infections except for antibiotic prophylaxis against *Pneumocystis carinii* and viruses, clinical immunologists were in favor for CLL patients (66.7%), whereas clinical hematologists reached a consensus against antibiotic prophylaxis in patients with CLL (76.5%), MM (70.6%) and lymphoma (76.5%). A consensus against the use of antibiotic prophylaxis in the presence of hypogammaglobulinemia was reached by both clinical hematologists and clinical immunologists. The results obtained in this section of the questionnaire are shown in the Supplementary Materials Table S2.

#### 3.2.3. Treatment with IVIG and Follow-Up

As shown in Table 4, there was a consensus against the use of IgRT after the baseline immunological evaluation in patients with CLL (68.6%), MM (77.1%) and lymphoma (80%). In patients with CLL, MM and lymphoma, no consensus was reached in favor of the use of IVIG if there are recurrent infections or if there is evidence of hypogammaglobulinemia. In patients who are candidates for IVIG therapy because of recurrent infections, all participants agreed on the use of doses of 400 mg/kg every 4 weeks over 12 months (88.6%), the maintenance of minimum IgG levels between 500 and 700 mg/dL (91.4%) and the monitoring of IgG levels to optimize the doses of IgG (77.1%). In patients treated with IVIG, there was a consensus regarding the monitoring of IgG levels (97.1%), every 3 months (70.6%), as well as to discontinue IVIG after the recovery of IgG levels (77.1%). Finally, both hematologists (68.7%) and clinical immunologists (100%) were aware of the new indications of IVIG therapy approved by the EMA for the management of SID. Detailed results of treatment with IVIG and the follow-up of patients receiving IVIG are shown in the Supplementary Materials Tables S3 and S4.

### 3.3. Recommendations

#### 3.3.1. Baseline Immunological Evaluation

Participants 100% agreed on the need to establish recommendations/guidelines for the management of immunodeficiencies in hematological malignancies (CLL, MM, lymphoma, HSCT); these would include an immunological evaluation in the initial patient’s assessment with a detailed medical history, a physical examination and total proteins and protein electrophoresis in patients with B-cell neoplasms, as well as the quantification of IgG, IgA and IgM levels and IgG subclasses when SID is suspected. There was also a consensus on the immunophenotyping of T, B and natural killer subpopulations (91.4%). In contrast, regarding the determination of specific antibodies against immunizations with protein and polysaccharide antigens, a consensus was achieved among immunologists (83%), but not among hematologists (53%). In the case of SID, patients should be managed by hematologists (66.7%) or immunologists (100%), or by both specialists (88.6%). Results obtained in the management of SID in patients with hematological malignancies are shown in the Supplementary Materials Table S5.

#### 3.3.2. Prophylaxis of Infection

There was a strong consensus (between 94.3% and 100%), independently of the participant’s specialty, regarding the recommendation of active immunization against seasonal influenza and H1N1 infection, pneumococcus and *Haemophilus influenzae* in patients with CLL, MM and lymphoma. The use of antibiotic prophylaxis after the baseline immunological evaluation in patients with CLL (94.1%), MM (94.1%) and lymphoma (88.2%) obtained a consensus among clinical hematologists only. In the case of hypogammaglobulinemia, a consensus on the use of antibiotic prophylaxis was not obtained. Detailed results are shown in the Supplementary Materials Table S6.

#### 3.3.3. Use of IVIG and Follow-Up

Participants, particularly clinical hematologists, agreed against the indication of IVIG therapy after the initial immunological evaluation. In the presence of recurrent or severe infections, clinical immunologists reached a consensus in favor of treatment with IVIG after antibiotic failure in patients with CLL (77.8%), MM (77.8%) and lymphoma (72.2%). In contrast, clinical hematologists reached a consensus against this, with percentages of 70.6%, 76.5% and 76.5%, respectively. In patients with lymphoma and in the presence of hypogammaglobulinemia, only clinical hematologists agreed (82.4%) on the use of IVIG. With respect to the implementation of treatment with IVIG, there was consistent agreement by all participants on the need to have available a clinical protocol for the use of IVIG in the management of SID (94.3%) and to monitor trough IgG levels to determine the correct dose of IVIG (85.7%). Detailed results are shown in the Supplementary Materials Table S7.

There was a consensus (97.2%) regarding the need for the monitoring of IgG levels in patients treated with IVIG, with a frequency of every 3 (82.7%) or 6 (77.1%) months, as well as to assess clinical efficacy every 3 (85.7%) or 6 (82.9%) months. There was a consensus against the withdrawal of IVIG therapy after completing chemotherapy (80%), but a consensus in favor of IVIG discontinuation after the recovery of IgG levels (77.1%). Results related to the follow-up and monitoring of patients treated with IVIG are shown in the Supplementary Materials Table S8. Final recommendations are summarized in Table 5.

## 4. Discussion

Although there has been a significant improvement in the treatment of several hematological malignancies, thanks to the introduction of targeted therapies and other new drugs, SID is a disease connected to solid tumors, lymphoproliferative and myeloproliferative disorders [25,26]. Immunodeficiency in these patients has an impact on the outcome, particularly on the risk of infection, which affects quality of life and is the major cause of morbidity and mortality [27]. The adequate management of these patients in routine daily practice is challenging for different reasons, including the lack of standardized protocols and disparities related to appropriate vaccination coverage and immunization schedules and the use of antibiotic prophylaxis. There are also uncertainties regarding when to initiate therapy or when to discontinue IgRT [28,29].

An international online survey of 230 physicians from seven countries involved in the diagnosis of SID and the prescription of IgRT in patients with hematological malignancies highlighted the need for harmonized, evidence-based diagnostic and treatment guidelines for SID in these patients [30]. Despite recommendations provided by different guidelines [13,14,31,32] regarding the administration of IVIG or subcutaneous immunoglobulin (SCIg) in patients with SID, there is a lack of information on how these recommendations are implemented in daily clinical practice when managing SID. In addition, the EMA guideline [14] highlights the relevance of functional studies of antibody production for IgRT indication beyond hypogammaglobulinemia [2,15]. This latter issue is especially relevant in MM patients, in which IgG may be high despite profound polyclonal hypogammaglobulinemia.

The present survey provides information on a large number of questions related to the assessment of SID in patients with hematological malignancies, as a baseline initial investigation when SID is suspected. It also provides information about treatment strategies, the prophylaxis of infections, follow-up and the type of monitoring of IVIG therapy. Questions on whether there were differences or coincidences between clinical hematologists and clinical immunologists were investigated. To our knowledge, this is the first survey of these characteristics carried out in Spain. Most of the participants worked in public hospitals and reported both the lack of and the need for specific protocols for the diagnosis and management of SID with IgRT. Importantly, participants reported that facilities for the performance of baseline immunological studies were already available in most of their own centers.

The questionnaire showed that there was a consensus on the need to perform immunological studies in patients with CLL, MM and lymphoma as BCLPD, as well as in HSCT recipients and in any patients diagnosed with other hematological malignancies and with a prior history of recurrent infection episodes. Surprisingly, a consensus regarding the need for baseline immunological evaluation in patients of advanced age or those who are fragile was not obtained. Similar consensus guidelines on IgRT have been proposed by the UK Primary Immunodeficiency Network and the British Society of Immunology based on a Delphi approach [33]. It should be noted that there were discrepancies related to the study of IgG subclasses and IgG antibody titers to previous immunizations/exposure, which were recommended by consensus only by clinical immunologists, indicating that hematologists do not perform such studies. In addition, clinical immunologists and hematologists reached a consensus on the study of lymphocyte subpopulations of T, B and NK cells. In this regard, there was a consensus on extending the immunological work-up to lymphocyte subsets, as recent data support the relevance of combined immune defects in the risk of severe infection and suggest its relevance also for cancer progression or recurrence [34]. Therefore, incorporating immunophenotypic assessment in SID can enhance disease characterization and guide the initiation of immunoglobulin replacement therapy. However, a discrepancy was found regarding the need for specific antibodies against immunizations with protein and polysaccharide antigens. These findings highlight the need to create standardized guidelines to improve current recommendations and guide future work in this field.

In relation to the prophylaxis of infections in hematological patients with malignancy, there was a high rate of agreement between hematologists and immunologists for active immunization against seasonal influenza, H1N1 and pneumococcus, and reduced agreement for *H. influenzae*. These findings agree with data reported in a recent systematic review and meta-analysis, in which vaccinations reduced the risk of clinically documented infections in patients with hematological malignancies by 63% [35]. In the use of antibiotic prophylaxis, an overall consensus was not obtained, although most clinical immunologists had an opinion against the use of antibiotics for the prophylaxis of infections, except in cases of recurrent infections (excluding *Pneumocystis carinii* and viruses). In this context, in a recent study in a large cohort of patients with MM and autologous bone marrow transplantation, the use of antibiotic prophylaxis was associated with a higher risk of multidrug-resistant bacteria with respect to colony growth factors [36].

On the other hand, regarding the indications for treatment with IVIG, there was a consensus on treating patients with SID and recurrent infection episodes, but not in the case of patients with IgG deficiency without infection, in line with the EMA indications and a recent meta-analysis [35].

There was a consensus between hematologists and immunologists on the starting and maintenance doses of IVIG as well monitoring IgG levels every 3 months, and to discontinue treatment after the recovery of IgG levels.

In relation to recommendations, there was consistent agreement on the need to have available a clinical protocol for the use of IVIG in the management of SID and to monitor trough IgG levels to determine the correct dose of IVIG. Both hematologists and clinical immunologists were aware of the new indications for IVIG therapy approved by the EMA [14] for the management of SID.

The fact that about 40% of the responses analyzed corresponded to fully completed questionnaires may be viewed as a limitation of the survey, but the number of hematologists and clinical immunologists was similar, which allowed a comparison of their opinions corresponding to these specialties. The influence of chemotherapeutic regimens or novel B-cell-targeting therapies, like CAR-T or bispecific antibodies, used to treat the different hematological malignancies and diseases was not analyzed. It should be noted that the Delphi questionnaire was designed and the survey was conducted during 2018–2020. At that time, these therapies were less prevalent in clinical practice. Additionally, the use of SCIg supplementation was not included in the Delphi survey as it was not a standard practice in the hematology field in Spain and limited data were available on the use of SCIg in the context of SID [37,38]. Given the growing adoption of these novel therapies, the use of SCIg supplementation as a replacement therapy in cases of SID associated with lymphoproliferative disorders is expected to expand. Reassessment of the survey in the forthcoming years will be necessary to track new treatment strategies, particularly for patients exhibiting pronounced decreases in immunoglobulin levels due to these therapies. Moreover, this survey does not reflect the change in mentality of clinical hematologists arising from the COVID-19 pandemic regarding the importance of infections. However, the extension of the questionnaire allowed the capture of salient aspects of the current clinical practice and the proposal of recommendations for SID in oncohematological patients.

Notably, the consensus found in this survey aligns closely with the recommendations outlined in existing guidelines, illustrating the significant coherence between clinical practice and established directives [13,14,28,31,32,33].

In summary, the findings of the present survey underline the need to promote the presence of clinical immunologists in Spanish hospitals, thereby fostering multidisciplinary collaboration with hematologists and improving the management of SID in patients with B-cell neoplasms in daily practice.

## Figures and Tables

**Table 1:** Demographic data of participants.

Data	Percentage
Years of professional practice	
<5	9
5–10	29
11–15	9
>15	54
Hospital characteristics	
Public hospital	94
Private hospital	6
<200 beds	6
200–400 beds	11
>400 beds	83
Center with immunology consultation	65
Center with immunology laboratory	75
Center with protocol for SID	35
Patients	
Center with hematological neoplasms and SID	100
0–10 patients/year	9
10–20 patients/year	32

SID: secondary immunodeficiency.

**Table 2:** Baseline evaluation at initial survey in patients with hematological malignancies (responses provided only by hematologists, n = 17).

Baseline Immunological Evaluation in the Initial Survey	Consensus	
	Against	In Favor
Patients with chronic lymphocytic leukemia (CLL)	0	100.0
Patients with multiple myeloma (MM)	0	100.0
Patients with lymphoma	0	100.0
Hematopoietic stem cell transplantation (HSCT) recipients	0	100.0
Patients of advanced age/fragile	47.1	52.9

**Table 3:** Overall responses in the section on prophylaxis in the management of secondary immunodeficiency (SID) in patients with hematological malignancies in clinical practice (responses provided by overall specialists, n = 35).

Prophylaxis of Infection	Consensus	
	Against	In Favor
How often do doctors think that patients with hematological malignancies receive active immunization against the following infections:		
Patients with CLL		
Seasonal influenza and H1N1	2.9	97.1
Pneumococcus	20.0	80.0
*Haemophilus influenzae*	51.4	48.6
Patients with MM		
Seasonal influenza and H1N1	8.6	91.4
Pneumococcus	25.7	74.3
*Haemophilus influenzae*	48.6	51.4
Patients with lymphoma		
Seasonal influenza and H1N1	8.6	91.4
Pneumococcus	28.6	71.4
*Haemophilus influenzae*	51.4	48.6
Except for prophylaxis against *Pneumocystis carinii* and viruses, how often do doctors think that patients receive antibiotic prophylaxis for recurrent infections in:		
Patients with CLL	54.3	45.7
Patients with MM	57.1	42.9
Patients with lymphoma	57.1	42.9
How often do you use antibiotic prophylaxis if there is evidence of hypogammaglobulinemia in:		
Patients with CLL	71.4	28.6
Patients with MM	74.3	25.7
Patients with lymphoma	74.3	25.7

CLL: chronic lymphocytic leukemia; MM: multiple myeloma.

**Table 4:** Overall responses regarding treatment with intravenous IgG (IVIG) in current clinical practice after recurrent infections or evidence of hypogammaglobulinemia in secondary immunodeficiency (SID) patients with hematological malignancies (responses provided by overall specialists, n = 35).

Use of IVIG	Consensus	
	Against	In Favor
How do you often use IVIG after baseline immunological evaluation?		
Patients with CLL	68.6	31.4
Patients with MM	77.1	22.9
Patients with lymphoma	80.0	20.0
How often do doctors use IVIG if there are recurrent infections in:		
Patients with CLL	51.4	48.6
Patients with MM	68.6	31.4
Patients with lymphoma	60.0	40.0
How often do doctors use IVIG if there is evidence of hypogammaglobulinemia in:		
Patients with CLL	55.9	44.1
Patients with MM	73.5	26.5
Patients with lymphoma	67.6	32.3

CLL: chronic lymphocytic leukemia; MM: multiple myeloma; IVIG: intravenous IgG.

**Table 5:** Summarized recommendations for patients with SID and hematological malignancies.

Recommendations		Consensus
**Baseline immunological evaluation**		
Guidelines are necessary for the management of immunodeficiencies in hematological patients		100%
In the initial survey of CLL, MM, lymphoma and HSCT recipients		100%
After recurrent/severe infection when SID is suspected in CLL, MM, lymphoma and HSCT recipients		100%
In patients with B-cell neoplasms (anamnesis, physical examination, proteins total/electrophoresis)		100%
Quantification of IgG, IgA and IgM levels when SID is suspected		100%
Quantification of IgG subclasses when SID is suspected		77%
Specific antibodies against immunization with protein and polysaccharide antigens when SID is suspected		83% †
Immunophenotyping subpopulations of T, B, natural killer when SID is suspected		91%
Chest CT scan in case of suspected SID		67% †
Auto-antibodies (antinuclear, anti-DNA, anti-phospholipid, anti-platelet, anti-neutrophil, etc.) in case of suspected SID		72% †
Functional immunological evaluation after recurrent and/or severe infection when SID is suspected	In patients with CLL	74%
	In patients with MM	77%
	In patients with lymphoma	78% †
**Prophylaxis of infection**		
Patients with CLL should receive active immunization against	Seasonal influenza and H1N1 and pneumococcus	97%
	*Haemophilus influenzae*	94%
	HAV and HBV (in sero-negative patients)	91%
Patients with MM should receive active immunization against	Seasonal influenza and H1N1 and pneumococcus	97%
	*Haemophilus influenzae*	94%
	HAV and HBV (in sero-negative patients)	91%
Patients with lymphoma should receive active immunization against	Seasonal influenza and H1N1	97%
	Pneumococcus	94%
	*Haemophilus influenzae*	91%
	HAV and HBV (in sero-negative patients)	86%
Antibiotic prophylaxis after baseline evaluation should be established	In patients with CLL	94% *
	In patients with MM	94% *
	In patients with lymphoma	88% *
Antibiotic prophylaxis in cases of recurrent infection (excluding *Pneumocystis carinii* and viruses) should be established	In patients with CLL	89% †
	In patients with MM	89% †
	In patients with lymphoma	89% †
**Use of intravenous IgG (IVIG)**		
If there are recurrent infections	In patients with CLL	78% †
	In patients with MM	78% †
	In patients with lymphoma	72% †
In the presence of hypogammaglobulinemia in patients with lymphoma		82% *
Requirement to have a clinical protocol for the management of IVIG in patients with SID		94%
Start treatment with IVIG at a dose of 400 mg/kg every 4 weeks for 12 months in the candidate patient		80%
Personalize the IVIG dose		91%
The aim of maintenance therapy is to maintain IgG trough levels between 500 and 700 mg/dL in patients with recurrent infections and malignant blood disease		94%
Early decision on IVIG replacement therapy to prevent the development or progression of bronchiectasis		91%
Need for monitoring of IgG levels to determine the correct dose of IVIG		86%
**Follow-up and monitoring of patients receiving IVIG therapy**		
Monitoring of IgG levels		97%
Monitoring of the clinical efficacy of IVIG (decrease in and/or absence of bacterial and viral infections)		97%
Monitoring of IgG levels	Every 3 months	83%
	Every 6 months	77%
Monitoring of the clinical efficacy of IVIG therapy	Every 3 months	86%
	Every 6 months	83%
Discontinuation of treatment with IVIG after recovery of IgG levels		77%

* Consensus achieved by hematologists; † consensus achieved by immunologists; CLL: chronic lymphocytic leukemia; MM: multiple myeloma; HSCT: hematopoietic stem cell transplantation; SID: secondary immunodeficiency; HAV: hepatitis A virus; HBV: hepatitis B virus; IVIG: intravenous IgG.

## Data Availability

Data are available from the authors upon request.

## Supplementary Materials

The following supporting information can be downloaded at: https://www.mdpi.com/article/10.3390/jcm12196356/s1, Table S1: Current clinical practice in the management of SID in patients with hematological malignancies: baseline immunological assessment; Table S2: Current clinical practice in the management of SID in patients with hematological malignancies: prophylaxis of infection; Table S3: Current clinical practice in the management of SID in patients with hematological malignancies: treatment with intravenous IgG (IVIG); Table S4: Current clinical practice in the management of SID in patients with hematological malignancies: follow-up and monitoring of patients receiving intravenous IgG (IVIG); Table S5: Recommendation guidelines for the management of SID in patients with hematological malignancies; Table S6: Recommendation guidelines for the prophylaxis of infections in patients with SID and hematological malignancies; Table S7: Recommendation guidelines for the use of intravenous IgG (IVIG) therapy in patients with SID and hematological malignancies; Table S8: Recommendation guidelines for the follow-up and monitoring of patients receiving intravenous IgG (IVIG) therapy.
