# Supplementary material for: Current Approach in the Management of Secondary Immunodeficiency in Patients with Hematological Malignancies: Spanish Expert Consensus Recommendations
Source: J Clin Med. 2023 Oct 4;12(19):6356. doi: 10.3390/jcm12196356 (PMC10573502; doi:10.3390/jcm12196356)
Supplement: Supplementary file 1 [file jcm-12-06356-s001.zip › jcm-2573132-supplementary.pdf]

SUPPLEMENTARY MATERIAL

Table S1. Current clinical practice in the management of SID in patients with hematological malignancies: baseline immunological assessment

Questions	4-point Likert scale, % of responses				Consensus		Mean
	1 = Never	2 = Sometimes	3 = Frequently	4 = Always	Against	In favor	
• Do you perform baseline immunological evaluation at your own center?							
Clinical hematologists, n = 17	0	0	23.5	76.5	0	100.0	3.76
Clinical immunologists, n = 18	5.6	16.7	22.2	55.6	22.2	77.8	3.28
Overall, n = 35	2.9	8.6	22.9	65.7	11.4	88.6	3.51
• Do you perform baseline immunological evaluation in the initial study of:							
Patients with chronic lymphocytic leukemia (CLL)							
Clinical hematologists, n = 17	0	0	11.8	88.2	0	100.0	3.88
Patients with multiple myeloma (MM)							
Clinical hematologists, n = 17	0	0	5.9	94.1	0	100.0	3.94
Patients with lymphoma							
Clinical hematologists, n = 17	0	0	35.3	64.7	0	100.0	3.65
Hematopoietic stem cell transplantation (HSCT) recipients							
Clinical hematologists, n = 17	0	0	17.7	82.3	0	100.0	3.82
Patients of advanced age/fragile							
Clinical hematologists, n = 17	0	47.1	47.1	5.9	47.1	52.9	2.59
• Frequency with which the following baseline immunological studies are performed in patients with B-cell malignancies in your center:							
Detailed anamnesis (personal/family history of recurrent/severe/unusual infections)							
Clinical hematologists, n = 17	0	17.7	23.5	58.8	17.6	82.4	3.41
Clinical immunologists, n = 18	0	11.1	11.1	77.8	11.1	89.0	3.67
Overall, n = 35	0	14.3	17.1	68.6	14.3	85.7	3.54
Complete physical examination (skin/mucosas)							
Clinical hematologists, n = 17	0	5.9	5.9	88.2	5.9	94.1	3.82
Clinical immunologists, n = 18	0	16.7	33.3	50.0	16.7	83.3	3.33
Overall, n = 35	0	11.4	20.0	68.6	11.4	88.6	3.57
Total proteins, protein electrophoresis							
Clinical hematologists, n = 17	0	0	11.8	88.2	0	100.0	3.88
Clinical immunologists, n = 18	0	0	27.8	72.2	0	100.0	3.72
Overall, n = 35	0	0	20.0	80.0	0	100.0	3.80
Quantification serum levels of IgG, IgA, IgM							
Clinical hematologists, n = 17	0	0	17.7	82.3	0	100.0	3.82
Clinical immunologists, n = 18	0	5.6	22.2	72.2	5.6	94.4	3.67
Overall, n = 35	0	2.9	20.0	77.1	2.9	97.1	3.74
• In case of suspicion of SID, indicate the frequency with which the following baseline immunological studies are performed in your center:							
Study of IgG subclasses							
Clinical hematologists, n = 17	23.5	52.9	11.8	11.8	76.5	23.5	2.12
Clinical immunologists, n = 18	0	27.8	5.6	66.7	27.8	72.2	3.39
Overall, n = 35	11.4	40.0	8.7	40.0	51.4	48.6	2.77
IgG antibody titers to previous immunizations/exposure							
Clinical hematologists, n = 17	35.3	41.2	23.5	0	76.5	23.5	1.88
Clinical immunologists, n = 18	0	22.2	27.8	50.0	22.2	77.8	3.28
Overall, n = 35	17.1	31.4	25.7	25.7	48.6	51.4	2.60
Specific antibodies against immunization with protein and polysaccharide antigen (tetanus toxoid, conjugated anti-pneumococcal and anti-tetanus, *S. typhi*, *Haemophilus influenza* b)							
Clinical hematologists, n = 17	35.3	41.2	23.5	0	76.5	23.5	1.88
Clinical immunologists, n = 18	5.6	27.8	22.2	44.4	33.3	66.7	3.06
Overall, n = 35	20.0	34.3	22.9	22.9	54.3	45.7	2.49
Immunophenotyping lymphocyte subpopulations T, B, natural killer							
Clinical hematologists, n = 17	17.6	35.3	23.5	23.5	52.9	47.1	2.53
Clinical immunologists, n = 18	0	16.7	22.2	61.1	16.7	83.3	3.44
Overall, n = 35	8.6	25.7	22.9	42.9	34.3	65.7	3.00
Thoracic computed tomography							
Clinical hematologists, n = 17	11.8	41.2	29.4	17.7	52.9	47.1	2.53
Clinical immunologists, n = 18	0	55.6	27.8	16.7	55.7	44.4	2.61
Overall, n = 35	5.7	48.6	28.6	17.1	54.3	45.7	2.57
Memory B-cells immunophenotyping							
Clinical hematologists, n = 17	41.2	35.3	5.9	17.7	76.5	23.5	2.00
Clinical immunologists, n = 18	5.6	44.4	22.2	27.8	50.0	50.0	2.72
Overall, n = 35	22.9	40.0	14.3	22.9	62.9	37.1	2.37

Table S2. Current clinical practice in the management of SID in patients with hematological malignancies: prophylaxis of infection

Questions	4-point Likert scale, % of responses				Consensus		Mean
	1 = Never	2 = Sometimes	3 = Frequently	4 = Always	Against	In favor	
• How often do you think that patients with chronic lymphocytic leukemia (CLL) receive active immunization against the following infections:							
Seasonal influenza and H1N1							
Clinical hematologists, n = 17	0	0	29.4	70.6	0	100.0	3.71
Clinical immunologists, n = 18	0	5.6	66.7	27.8	5.6	94.4	3.22
Overall, n = 35	0	2.9	48.6	48.6	2.9	97.1	3.46
Pneumococcus							
Clinical hematologists, n = 17	0	0	35.9	64.7	0	100.0	3.65
Clinical immunologists, n = 18	0	38.9	33.3	27.8	38.9	61.1	2.89
Overall, n = 35	0	20.0	34.3	45.7	20.0	80.0	3.26
Haemophilus influenzae							
Clinical hematologists, n = 17	0	52.9	29.4	17.6	52.9	47.1	3.65
Clinical immunologists, n = 18	5.6	44.4	44.4	5.6	50.0	50.0	2.50
Overall, n = 35	2.9	48.6	37.1	11.4	51.4	48.6	2.57
• How often do you think that patients with MM receive active immunization against the following infections:							
Seasonal influenza and H1N1							
Clinical hematologists, n = 17	0	5.9	23.5	70.6	5.9	94.1	3.65
Clinical immunologists, n = 18	0	11.1	72.2	16.7	11.1	88.9	3.06
Overall, n = 35	0	8.6	48.6	42.9	8.6	91.4	3.34
Pneumococcus							
Clinical hematologists, n = 17	0	11.8	23.5	64.7	11.8	88.2	3.53
Clinical immunologists, n = 18	0	38.9	33.3	27.8	38.9	61.1	2.89
Overall, n = 35	0	25.7	28.6	45.7	25.7	74.3	3.20
Haemophilus influenzae							
Clinical hematologists, n = 17	0	52.9	23.5	23.5	52.9	47.1	2.71
Clinical immunologists, n = 18	5.6	38.9	44.4	11.1	44.4	55.6	2.61
Overall, n = 35	2.9	45.7	34.3	17.1	48.6	51.4	2.66
• How often do you think that patients with lymphoma receive active immunization against the following infections:							
Seasonal influenza and H1N1	0	5.9	29.4	64.7	5.9	94.1	3.59
Clinical hematologists, n = 17	0	11.1	61.1	27.8	11.1	88.9	3.17
Clinical immunologists, n = 18	0	8.6	45.7	45.7	8.6	91.4	3.37
Overall, n = 35							
Pneumococcus							
Clinical hematologists, n = 17	5.9	11.8	35.3	47.1	17.6	82.3	3.24
Clinical immunologists, n = 18	0	38.9	38.9	22.2	38.9	61.1	2.83
Overall, n = 35	2.9	25.7	37.1	34.3	28.6	71.4	3.03
Haemophilus influenzae							
Clinical hematologists, n = 17	11.8	47.1	35.3	5.9	58.8	41.2	2.35
Clinical immunologists, n = 18	5.6	38.9	50.0	5.6	44.4	55.6	2.56
Overall, n = 35	8.6	42.9	42.9	5.7	51.4	48.6	2.46
• Except for prophylaxis against *Pneumocystis carinii* and viruses, how often do you use antibiotic prophylaxis if there are recurrent infections in:							
Patients with chronic lymphocytic leukemia (CLL)							
Clinical hematologists, n = 17	11.8	64.7	23.5	0	76.5	23.5	2.12
Clinical immunologists, n = 18	0	33.3	55.6	11.1	33.3	66.7	2.78
Overall, n = 35	5.7	48.6	40.0	5.7	54.3	45.7	2.46
Patients with multiple myeloma (MM)							
Clinical hematologists, n = 17	17.6	52.9	23.5	5.9	70.6	29.4	2.18
Clinical immunologists, n = 18	0	44.4	44.4	11.1	44.4	55.6	2.67
Overall, n = 35	8.6	48.6	34.3	8.6	57.1	42.9	2.43
Patients with lymphoma							
Clinical hematologists, n = 17	23.5	52.9	23.5	0	76.5	23.5	2.00
Clinical immunologists, n = 18	0	38.9	50.0	11.1	38.9	61.1	2.72
Overall, n = 35	11.4	45.7	37.1	5.7	57.1	42.9	2.37
• How often do you use antibiotic prophylaxis if there is evidence of hypogammaglobulinemia in:							
Patients with chronic lymphocytic leukemia (CLL)							
Clinical hematologists, n = 17	47.1	35.3	17,6	0	82.3	17.6	1.71
Clinical immunologists, n = 18	5.6	55.6	27.8	11.1	61.1	38.9	2.44
Overall, n = 35	25.7	45.7	22.9	5.67	71.4	28.6	2.09
Patients with multiple myeloma (MM)							
Clinical hematologists, n = 17	47.1	35.3	17.6	0	82.3	17.6	1.71
Clinical immunologists, n = 18	0	66,7	22.2	11.1	66.7	33.3	2.44
Overall, n = 35	22.9	51.4	20.0	5.7	74.3	25.7	2.09
Patients with lymphoma							
Clinical hematologists, n = 17	47.1	35.3	17.6	0	82.3	17.6	1.71
Clinical immunologists, n = 18	0	66.7	22.2	11.1	66.7	33.3	2.44
Overall, n = 35	22.9	51.4	20.0	5.7	74.3	25.7	2.09

Table S3. Current clinical practice in the management of SID in patients with hematological malignancies: treatment with intravenous IgG (IVIG)

Questions	4-point Likert scale, % of responses				Consensus		Mean
	1 = Never	2 = Sometimes	3 = Frequently	4 = Always	Against	In favor	
• How often do you use IVIG after baseline immunological evaluation in:							
Patients with chronic lymphocytic leukemia (CLL)							
Clinical hematologists, n = 17	23.5	47.1	17.6	11.8	70.6	29.4	2.18
Clinical immunologists, n = 18	5.6	61.1	16.7	16.7	66.7	33.3	2.44
Overall, n = 35	14.3	54.3	17.1	14,3	68.6	31.4	2.13
Patients with multiple myeloma (MM)							
Clinical hematologists, n = 17	35.3	52.9	5.9	5.9	88.2	11.8	1.82
Clinical immunologists, n = 18	5.6	61.1	22.2	11.1	66.7	33.3	2.39
Overall, n = 35	20.0	57.1	14.3	8.6	77.1	22.9	2.11
Patient with lymphoma							
Clinical hematologists, n = 17	35.3	58.8	0	5.9	94.1	5.9	1.76
Clinical immunologists, n = 18	5.6	61.1	16.7	16.7	66.7	33.3	2.44
Overall, n = 35	20.0	60.0	8.6	11.4	80.0	20.0	2.11
• How often do you use IVIG if there are recurrent infections in:							
Patients with chronic lymphocytic leukemia (CLL)							
Clinical hematologists, n = 17	5.9	35.3	47.1	11.8	41.2	58.8	2.65
Clinical immunologists, n = 18	0	61.1	16.7	22.2	61.1	38.9	2.61
Overall, n = 35	2.9	48.6	31.4	17.1	51.4	48.6	2.63
Patients with multiple myeloma (MM)							
Clinical hematologists, n = 17	17.6	58.8	11.8	11.8	76.5	23.5	2.18
Clinical immunologists, n = 18	0	61.1	16.7	22.2	61.1	38.9	2.61
Overall, n = 35	8.6	60.0	14.3	17.1	68.6	31.4	2.40
Patient with lymphoma							
Clinical hematologists, n = 17	11.8	47.1	41.2	0	58.8	41.2	2.29
Clinical immunologists, n = 18	0	61.1	27.8	11.1	61.1	38.9	2.50
Overall, n = 35	5.7	54.3	34.3	5.7	60.0	40.0	2.40
• How often do you use IVIG if there is evidence of hypogammaglobulinemia in:							
Patients with chronic lymphocytic leukemia (CLL)							
Clinical hematologists, n = 17	25.0	31.2	37.5	6.2	56.2	43.7	2.25
Clinical immunologists, n = 18	5.6	50.0	33.3	11.1	55.6	44.4	2.50
Overall, n = 35	14.7	41.2	35.3	8.8	55.9	44.1	2.38
Patients with multiple myeloma (MM)							
Clinical hematologists, n = 17	37.5	50.0	6.2	6.2	87.5	12.5	1.81
Clinical immunologists, n = 18	5.6	55.6	27.8	11.1	61.1	38.9	2.44
Overall, n = 35	20.6	52.9	17.6	8.8	73.5	26.5	2.15
Patients with lymphoma							
Clinical hematologists, n = 17	31.2	56.2	12.5	0	87.5	12.5	1.81
Clinical immunologists, n = 18	5.6	44.4	38.9	11.1	50.0	50.0	2.56
Overall, n = 35	17.6	50.0	26.5	5.9	67.6	32.3	2.21
• When you start IVIG, do you use it at doses of 400 mg/kg every 4 weeks for 12 months in the patient who is candidate to this treatment?							
Clinical hematologists, n = 17	0	11.8	64.7	23.5	11.8	88.2	3.12
Clinical immunologists, n = 18	0	11.1	66.7	22.2	11.1	88.9	3.11
Overall, n = 35	0	11.4	65.7	22.9	11.4	88.6	3.11
• Do you consider that the goal of maintenance therapy is to maintain minimum IgG levels between 500 mg/dL and 700 mg/dL in patients with recurrent infections and malignant blood disease? (individualizing doses later for each patient)							
Clinical hematologists, n = 17	0	0	52.9	47.1	0	100.0	3.47
Clinical immunologists, n = 18	5.6	11.1	38.9	44.4	16.7	83.3	3.22
Overall, n = 35	2.9	5.7	45.7	45.7	8.6	91.4	3.34
• Do you monitor trough IgG levels to determine the correct IVIG dose?							
Clinical hematologists, n = 17	17.6	23.5	11.8	47.1	41.2	58.8	2.88
Clinical immunologists, n = 18	0	5.6	11.1	83.3	5.6	94.4	3.78
Overall, n = 35	8.6	14.3	11.4	65.7	22.9	77.1	3.34

Table S4. Current clinical practice in the management of SID in patients with hematological malignancies: follow-up and monitoring of patients receiving intravenous IgG (IVIG)

**Questions**	4-point Likert scale, % of responses				Consensus		Mean
	1 = Never	2 = Sometimes	3 = Frequently	4 = Always	Against	In favor	
• During the follow-up of patients receiving IVIG, do you consider that should be performed:							
Monitoring of IgG levels							
Clinical hematologists, n = 17	0	5.9	29.4	64.7	5.9	94.1	3.59
Clinical immunologists, n = 18	0	0	16.7	83.3	0	100.0	3.83
Overall, n = 35	0	2.9	22.9	74.3	2.9	97.1	3.71
• How often do you perform monitoring of IgG levels?							
Every 3 months							
Clinical hematologists, n = 16	0	25.0	31.2	43.7	25.0	75.0	3.19
Clinical immunologists, n = 18	5.6	27.8	22.2	44.4	33.3	66.7	3.06
Overall, n = 34	2.9	26.5	26.5	44.1	29.4	70.6	3.12
Every 6 months							
Clinical hematologists, n = 16	12.5	43.7	31.2	12.5	56.2	43.7	2.44
Clinical immunologists, n = 18	5.6	38.9	22.2	33.3	44.4	55.6	2.83
Overall, n = 34	8.8	41.2	26.5	23.5	50.0	50.0	2.65
Every 12 months							
Clinical hematologists, n = 16	50.0	31.2	0	18.7	81.2	18.7	1.88
Clinical immunologists, n = 18	33.3	44.4	0	22.2	77.8	22.2	2.11
Overall, n = 34	41.2	38.2	0	20.6	79.4	20.6	2.00
• How often do you stop treatment with IgG in the following situations:							
After recovery of IgG levels							
Clinical hematologists, n = 17	0	11.8	23.5	64.7	11.8	88.2	3.53
Clinical immunologists, n = 18	11.1	22.2	33.3	33.3	33.3	66.7	2.89
Overall, n = 35	5.7	17.1	28.6	48.6	22.9	77.1	3.20

Table S5. Recommendation for the management of SID in patients with hematological malignancies

Questions	4-point Likert scale, % of responses				Consensus		Mean
	1 = Not necessary	2 = Optional	3 = Recommendable	4 = Indispensable	Against	In favor	
• Do you consider that guidelines/consensus are necessary in routine clinical practice for the management of immunodeficiencies in the hematological patient?							
Clinical hematologists, n = 17	0	0	29.4	70.6	0	100.0	3.71
Clinical immunologists, n = 18	0	0	27.8	72.2	0	100.0	3.72
Overall, n = 35	0	0	28.6	71.4	0	100.0	3.71
• Do you consider that baseline immunological evaluation should be carried out at the same center?							
Clinical hematologists, n = 17	0.0	5.9	52.9	41.2	5.9	94.1	3.35
Clinical immunologists, n = 18	0.0	0.0	61.1	38.9	0.0	100.0	3.39
Overall, n = 35	0.0	2.9	57.1	40.0	2.9	97.1	3.37
• Do you consider that baseline immunological evaluation should be referred to an external laboratory?							
Clinical hematologists, n = 17	41.2	52.9	5.9	0.0	94.1	5.9	1.65
Clinical immunologists, n = 18	33.3	61.1	5.6	0.0	94.4	5.6	1.72
Overall, n = 35	37.1	57.1	5.7	0.0	94.3	5.7	1.69
• Recommendation for a baseline immunological evaluation in the initial study of:							
Patients with chronic lymphocytic leukemia (CLL)							
Clinical hematologists, n = 17	0	0	11.8	88.2	0	100.0	3.88
Patients with multiple myeloma (MM)							
Clinical hematologists, n = 17	0	0	29.4	70.6	0	100.0	3.71
Patients with lymphoma							
Clinical hematologists, n = 17	0	0	29.4	70.6	0	100.0	3.71
Hematopoietic stem cell transplant (HSCT) recipients							
Clinical hematologists, n = 17	0	0	23.5	76.5	0	100.0	3.76
Patients of advanced age/fragile							
Clinical hematologists, n = 17	0	47.1	29.4	23.5	47.1	52.9	2.76
• Recommendation for a baseline immunological evaluation after appearance of recurrent or severe infections in case of suspicion of SID in:							
Patients with chronic lymphocytic leukemia (CLL)							
Clinical hematologists, n = 17	0	0	5.9	94.1	0	100.0	3.94
Patients with multiple myeloma (MM)							
Clinical hematologists, n = 17	0	0	5.9	94.1	0	100.0	3.94
Patients with lymphoma							
Clinical hematologists, n = 17	0	0	11.8	88.2	0	100.0	3.88
Hematopoietic stem cell transplant (HSCT) recipients							
Clinical hematologists, n = 17	0	0	5.9	94.1	0	100.0	3.94
Patients of advanced age/fragile							
Clinical hematologists, n = 17	0	0	47.1	52.9	0	100.0	3.53
• Recommendation for a baseline immunological study in patients with B-cell neoplasms							
Detailed anamnesis, personal/family history recurrent/severe/unusual infections							
Clinical hematologists, n = 17	0	0	23.5	76.5	0	100.0	3.76
Clinical immunologists, n = 18	0	0	5.6	94.4	0	100.0	3.94
Overall, n = 35	0	0	14.3	85.7	0	100.0	3.86
Complete physical examination (skin/mucosas)							
Clinical hematologists, n = 17	0	0	5.6	94.1	0	100.0	3.94
Clinical immunologists, n = 18	0	0	16.7	83.3	0	100.0	3.83
Overall, n = 35	0	0	11.4	88.6	0	100.0	3.89
Total proteins and protein electrophoresis							
Clinical hematologists, n = 17	0	0	17.6	82.3	0	100.0	3.82
Clinical immunologists, n = 18	0	0	11.1	88.9	0	100.0	3.89
Overall, n = 35	0	0	14.3	85.7	0	100.0	3.86
• In case of suspicion of SID, recommended baseline immunological evaluation should include							
Quantification of IgG, IgA and IgM levels							
Clinical hematologists, n = 17	0	0	11.8	88.2	0	100.0	3.88
Clinical immunologists, n = 18	0	0	0	100.0	0	100.0	4.00
Overall, n = 35	0	0	5.7	94.3	0	100.0	3.94
IgG subclasses							

Clinical hematologists, n = 17	0	0	35.3	35.3	29.4	70.6	3.00
Clinical immunologists, n = 18	0	0	38.9	44.4	16.7	83.3	3.22
Overall, n = 35	0	0	37.1	40.0	22.9	77.1	3.11
Spirometry							
Clinical hematologists, n = 17	0.0	47.1	41.2	11.8	47.1	52.9	2.65
Clinical immunologists, n = 18	0.0	16.7	44.4	38.9	16.7	83.3	3.22
Overall, n = 35	0.0	31.4	42.9	25.7	31.4	68.6	2.94
Isohemagglutinin titers							
Clinical hematologists, n = 17	5.9	70.6	23.5	0.0	76.5	23.5	2.18
Clinical immunologists, n = 18	5.6	44.4	22.2	27.8	50.0	50.0	2.72
Overall, n = 35	5.7	57.1	22.9	14.3	62.9	37.1	2.46
IgG antibody titers to previous immunizations/exposures							
Clinical hematologists, n = 17	0.0	29.4	52.9	17.7	29.4	70.6	2.88
Clinical immunologists, n = 18	0.0	11.1	22.2	66.7	11.1	88.9	3.56
Overall, n = 35	0.0	20.0	37.1	42.9	20.0	80.0	3.23
Specific antibodies against immunization with protein and polysaccharide antigen (tetanus toxoid, conjugated anti-pneumococcal and anti-tetanus, *S. typhi*, *Haemophilus influenza* b)							
Clinical hematologists, n = 17	0	47.1	29.4	23.5	47.0	52.9	2.76
Clinical immunologists, n = 18	0	16.7	11.1	72.2	16.7	83.3	3.56
Overall, n = 35	0	31.4	20.0	48.6	31.4	68.6	3.17
Immunophenotyping subpopulations T, B, natural killer							
Clinical hematologists, n = 17	5.9	5.9	52.9	35.3	11.8	88.2	3.18
Clinical immunologists, n = 18	0	5.6	33.3	61.1	5.4	94.4	3.56
Overall, n = 35	2.9	5.7	42.9	48.6	8.6	91.4	3.37
Thoracic computed tomography							
Clinical hematologists, n = 17	0	42.2	52.9	5.9	41.2	58.8	2.65
Clinical immunologists, n = 18	0	33.3	27.8	38.9	33.3	66.7	3.06
Overall, n = 35	0	37.1	40.0	22.9	37.1	62.9	2.86
B-cell memory immunophenotype							
Clinical hematologists, n = 17	11.8	41.2	29.4	17.7	52.9	47.1	2.53
Clinical immunologists, n = 18	0.0	5.6	50.0	44.4	5.6	94.4	3.39
Overall, n = 35	5.7	22.9	40.0	31.4	28.6	71.4	2.97
Auto-antibodies: anti-nuclear, anti-DNA, anti-phospholipid, anti-platelet, anti-neutrophil, etc							
Clinical hematologists, n = 17	5.9	47.1	23.5	23.5	52.9	47.1	2.65
Clinical immunologists, n = 18	0.0	27.8	50.0	22.2	27.8	72.2	2.94
Overall, n = 35	2.9	37.1	37.1	22.9	40.0	60.0	2.80
• Understanding functional immunological evaluation as the study of specific antibody production in response to vaccines (conjugated pneumococcal and/or tetanus, and/or *S. Typhi*, and/or *Haemophilus influenza b*). Indicate if, following the occurrence of recurrent and/or severe infections suspected to be SID, this study should be performed on patients prior to treatment							
Patients with CLL							
Clinical hematologists, n = 17	5.9	23.5	58.8	11.8	29.4	70.6	2.76
Clinical immunologists, n = 18	5.6	16.7	22.2	55.6	22.2	77.8	3.28
Overall, n = 35	5.7	20.0	40.0	34.3	25.7	74.3	3.03
Patients with MM							
Clinical hematologists, n = 17	5.9	17.7	64.7	11.8	23.5	76.5	2.82
Clinical immunologists, n = 18	5.6	16.7	22.2	55.6	22.2	77.8	3.28
Overall, n = 35	5.7	17.1	42.9	34.3	22.9	77.1	3.06
Patients with lymphoma							
Clinical hematologists, n = 17	5.9	35.3	52.9	5.9	41.2	58.8	2.59
Clinical immunologists, n = 18	5.6	16.7	22.2	55.6	22.2	77.8	3.28
Overall, n = 35	5.7	25.7	37.1	31.4	31.4	68.6	2.94
• In case of SID, indicate your recommendation level on who should manage the patient							
The clinical hematologist							
Clinical hematologists, n = 17	0.0	35.3	17.7	47.1	35.3	64.7	3.12
Clinical immunologists, n = 18	0.0	33.3	27.8	38.9	33.3	66.7	3.06
Overall, n = 35	0.0	34.3	22.9	42.9	34.3	65.7	3.09
The clinical immunologist							
Clinical hematologists, n = 17	0.0	35.3	41.2	23.5	35.3	64.7	2.88
Clinical immunologists, n = 18	0.0	0.0	44.4	55.6	0.0	100.0	3.56
Overall, n = 35	0.0	17.1	42.9	40.0	17.1	82.9	3.23
Both (clinical hematologist and immunologist)							
Clinical hematologists, n = 17	5.9	17.7	47.1	29.4	23.5	76.5	3.00
Clinical immunologists, n = 18	0.0	0.0	61.1	38.9	0.0	100.0	3.39
Overall, n = 35	2.9	8.6	54.3	34.3	11.4	88.6	3.20

Table S6. Recommendation for the prophylaxis of infections in patients with SID and hematological malignancies

Questions	4-point Likert scale, % of responses				Consensus		Mean
	1 = Not necessary	2 = Optional	3 = Recommendable	4 = Indispensable	Against	In favor	
• Indicate if patients with chronic lymphocytic leukemia (CLL) should receive active immunizations against:							
Seasonal influenza and H1N1							
Clinical hematologists, n = 17	0	0	0	100.0	0	100.0	4.00
Clinical immunologists, n = 18	0	5.6	22.2	72.2	5.6	94.4	3.67
Overall, n = 35	0	2.9	11.4	85.7	2.9	97.1	3.83
Pneumococcus							
Clinical hematologists, n = 17	0	0	0	100-0	0	100.0	4.00
Clinical immunologists, n = 18	0	5.6	27.8	66.7	5.6	94.4	3.61
Overall, n = 35	0	2.9	14.3	82.9	2.9	97.1	3.80
Haemophilus influenzae							
Clinical hematologists, n = 17	0	0	47.1	52.9	0	100.0	3.53
Clinical immunologists, n = 18	0	11.1	44.4	44.4	11.1	88.9	3.33
Overall, n = 35	0	5.7	45.7	48.6	5.7	94.3	3.43
HAV and HBV (in seronegative patients)							
Clinical hematologists, n = 17	0.0	5.9	58.8	35.3	5.9	94.1	3.29
Clinical immunologists, n = 18	5.6	5.6	50.0	38.9	11.1	88.9	3.22
Overall, n = 35	2.9	5.7	54.3	37.1	8.6	91.4	3.36
• Indicate if patients with multiple myeloma (MM) should receive active immunizations against:							
Seasonal influenza and H1N1							
Clinical hematologists, n = 17	0	0	0	100.0	0	100.0	4.00
Clinical immunologists, n = 18	0	5.6	22.2	72.2	5.6	94.4	3.67
Overall, n = 35	0	2.9	11.4	85.7	2.9	97.1	3.83
Pneumococcus							
Clinical hematologists, n = 17	0	0	5.9	94.1	0	100.0	3.94
Clinical immunologists, n = 18	0	5.6	27.8	66.8	5.6	94.4	3.61
Overall, n = 35	0	2.9	17.1	80.0	2.9	97.1	3.77
Haemophilus influenzae							
Clinical hematologists, n = 17	0	0	41.2	58.8	0	100.0	3.59
Clinical immunologists, n = 18	0	11.1	44.4	44.4	11.1	88.9	3.33
Overall, n = 35	0	5.7	42.9	51.4	5.7	94.3	3.46
HAV and HBV (in seronegative patients)	0.0	5.9	47.1	47.1	5.9	94.1	3.41
Clinical hematologists, n = 17	5.6	5.6	55.6	33.3	11.1	88.9	3.17
Clinical immunologists, n = 18	2.9	5.7	51.4	40.0	8.6	91.4	3.29
Overall, n = 35							
• Indicate if patients with lymphoma should receive active immunizations against:							
Seasonal influenza and H1N1							
Clinical hematologists, n = 17	0	0	5.9	94.1	0	100.0	3.94
Clinical immunologists, n = 18	0	5.6	22.2	72.2	5.6	94.4	3.67
Overall, n = 35	0	2.9	14.3	82.9	2.9	97.1	3.80
Pneumococcus							
Clinical hematologists, n = 17	0	5.9	5.9	88.2	5.9	94.1	3.82
Clinical immunologists, n = 18	0	5.6	33.3	61.1	5.6	94.4	3.56
Overall, n = 35	0	5.7	20.0	74.3	5.7	94.3	3.69
Haemophilus influenzae							
Clinical hematologists, n = 17	0	5.9	35.3	58.8	5.9	94.1	3.53
Clinical immunologists, n = 18	0	11.1	50.0	38.9	11.1	88.9	3.28
Overall, n = 35	0	8.6	42.9	48.6	8.6	91.4	3.40
HAV and HBV (in seronegative patients)							
Clinical hematologists, n = 17	0.0	17.7	47.1	35.3	17.7	82.4	3.18
Clinical immunologists, n = 18	5.6	5.6	55.6	33.3	11.1	88.9	3.17
Overall, n = 35	2.9	11.4	51.4	34.3	14.3	85.7	3.17
• Indicate if antibiotic prophylaxis should be established after baseline immunological evaluation in:							
Patients with chronic lymphocytic leukemia (CLL)							
Clinical hematologists, n = 17	0	5.9	17.6	76.5	5.9	94.1	3.71
Clinical immunologists, n = 18	0	61.1	16.7	22.2	61.1	38.9	2.61
Overall, n = 35	0	34.3	17.1	48.6	34.3	65.7	3.14
Patients with multiple myeloma (MM)							
Clinical hematologists, n = 17	0	5.9	29.4	64.7	5.9	94.1	3.59
Clinical immunologists, n = 18	0	61.1	16.7	22.2	61.1	38.9	2.61
Overall, n = 35	0	34.3	22.9	42.9	34.3	65.7	3.09
Patients with lymphoma							
Clinical hematologists, n = 17	0	11.8	35.3	52.9	11.8	88.2	3.41
Clinical immunologists, n = 18	0	50.0	27.8	22.2	50.0	50.0	2.72
Overall, n = 35	0	31.4	31.4	37.1	31.4	68.6	3.06
• Excluding prophylaxis for *Pneumocystis carinii* and							

viruses, do you believe that a therapeutic approach should be adopted through antibiotic prophylaxis if recurrent infections occur in:							
Patients with chronic lymphocytic leukemia (CLL)							
Clinical hematologists, n = 16	31.3	25.0	37.5	6.3	56.3	43.8	2.19
Clinical immunologists, n = 18	0.0	11.1	55.6	33.3	11.1	88.9	3.22
Overall, n = 34	14.7	17.7	47.1	20.6	32.4	67.7	2.74
Patients with multiple myeloma (MM)							
Clinical hematologists, n = 16	18.8	37.5	31.3	12.5	56.3	43.8	2.38
Clinical immunologists, n = 18	0.0	11.1	55.6	33.3	11.1	88.9	3.22
Overall, n = 34	8.8	23.5	44.1	23.5	32.4	67.7	2.82
Patients with lymphoma							
Clinical hematologists, n = 16	31.3	43.8	12.5	12.5	75.0	25.0	2.06
Clinical immunologists, n = 18	0.0	11.1	55.6	33.3	11.1	88.9	3.22
Overall, n = 34	14.7	26.5	35.3	23.5	41.2	58.8	2.68
• Indicate if antibiotic prophylaxis should be established in case of evidence of hypogammaglobinemia in:							
Patients with chronic lymphocytic leukemia (CLL)							
Clinical hematologists, n = 17	11.8	47.1	29.4	11.8	58.8	41.2	2.41
Clinical immunologists, n = 18	0	50.0	22.2	27.8	50.0	50.0	2.78
Overall, n = 35	5.7	48.6	25.7	20.0	54.3	45.7	2.60
Patients with multiple myeloma (MM)							
Clinical hematologists, n = 17	5.9	52.9	29.4	11.8	58.8	41.2	2.47
Clinical immunologists, n = 18	0	50.0	22.2	27.8	50.0	50.0	2.78
Overall, n = 35	2.9	51.4	25.7	20.0	54.3	45.7	2.63
Patients with lymphoma							
Clinical hematologists, n = 17	11.8	58.8	23.5	5.9	70.6	29.4	2.24
Clinical immunologists, n = 18	0	50.0	22.2	27.8	50.0	50.0	2.78
Overall, n = 35	5.7	54.3	22.9	17.4	60.0	40.0	2.51

Table S7. Recommendation for the use intravenous IgG (IVIG) therapy in patients with SID and hematological malignancies

Questions	4-point Likert scale, % of responses				Consensus		Mean
	1 = Not necessary	2 = Optional	3 = Recommendable	4 = Indispensable	Against	In favor	
• Treatment with IVIG should be indicated after initial immunological evaluation in:							
Patients with chronic lymphocytic leukemia (CLL)							
Clinical hematologists, n = 17	41.2	41.2	17.7	0	82.3	17.6	1.76
Clinical immunologists, n = 18	5.7	50.0	16.7	27.8	55.6	44.4	2.67
Overall, n = 35	22.9	45.7	17.1	14.3	68.6	31.4	2.23
Patients with multiple myeloma							
Clinical hematologists, n = 17	35.3	52.9	5.9	5.9	88.2	11.8	1.82
Clinical immunologists, n = 18	5.6	50.0	16.7	27.8	55.6	44.4	2.67
Overall, n = 35	20.0	51.4	11.4	17.1	71.4	28.6	2.26
Patients with lymphoma							
Clinical hematologists, n = 17	41.2	52.9	0	5.9	94.1	5.9	1.71
Clinical immunologists, n = 18	5.7	50.0	22.2	22.2	55.6	44.4	2.61
Overall, n = 35	22.9	51.4	11.4	14.3	74.3	25.7	2.17
• Treatment with IVIG should be indicated if there are recurrent infections in:							
Patients with chronic lymphocytic leukemia (CLL)							
Clinical hematologists, n = 17	29.4	41.2	17.6	11.8	70.6	29.4	2.12
Clinical immunologists, n = 18	0	22.2	38.9	38.9	22.2	77.8	3.17
Overall, n = 35	14.3	31.4	28.6	25.7	45.7	54.3	2.66
Patients with multiple myeloma							
Clinical hematologists, n = 17	35.3	41.2	17.6	5.9	76.5	23.5	1.94
Clinical immunologists, n = 18	0	22.2	38.9	38.9	22.2	77.8	3.17
Overall, n = 35	17.1	31.4	28.6	22.9	48.6	51.4	2.57
Patients with lymphoma							
Clinical hematologists, n = 17	35.3	41.2	23.5	0	76.5	23.5	1.88
Clinical immunologists, n = 18	0	27.8	33.3	38.9	27.8	72.2	3.11
Overall, n = 35	17.1	34.3	28.6	20.0	51.4	48.6	2.51
• Indicate if treatment with IVIG if there is evidence of hypogammaglobulinemia in:							
Patients with chronic lymphocytic leukemia (CLL)							
Clinical hematologists, n = 17	5.9	47.1	0	47.1	52.9	47.1	2.88
Clinical immunologists, n = 18	0	55.6	16.7	27.8	55.6	44.4	2.72
Overall, n = 35	2.9	51.4	8.6	37.1	54.3	45.7	2.80
Patients with multiple myeloma							
Clinical hematologists, n = 17	11.7	23.5	41.2	23.5	35.3	64.7	2.76
Clinical immunologists, n = 18	5.6	44.4	22.2	27.8	50.0	50.0	2.72
Overall, n = 35	8.6	34.3	31.4	25.7	42.9	57.1	2.74
Patients with lymphoma							
Clinical hematologists, n = 17	5.9	11.8	58.8	23.5	17.6	82.4	3.00
Clinical immunologists, n = 18	5.9	50.0	16.7	27.8	55.6	44.4	2.67
Overall, n = 35	5.7	31.4	37.1	25.7	37.1	62.9	2.83
• Do you consider that all centers should have a clinical protocol available for the management with IVIG in patients with SID?							
Clinical hematologists, n = 17	0	5.9	23.5	70.6	5.9	94.1	3.65
Clinical immunologists, n = 18	0	5.6	38.9	55.6	5.6	94.4	3.50
Overall, n = 35	0	5.7	31.4	62.9	5.7	94.3	3.57
• Do you consider that IVIG treatment should be administered at a starting dose of 400 mg/kg every 4 weeks for 12 months in the candidate patient?							
Clinical hematologists, n = 17	5.9	5.9	41.2	47.1	11.8	88.2	3.29
Clinical immunologists, n = 18	0.0	27.8	61.1	11.1	27.8	72.2	2.83
Overall, n = 35	2.9	17.1	51.4	28.6	20.0	80.0	3.06
• Do you consider that the IVIG dosage should be personalized?							
Clinical hematologists, n = 17	11.8	5.9	41.2	41.2	17.7	82.4	3.12
Clinical immunologists, n = 18	0.0	0.0	27.8	72.2	0.0	100.0	3.72
Overall, n = 35	5.7	2.9	34.3	57.1	8.6	91.4	3.43
• Do you consider that the aim of maintenance therapy is to maintain minimum IgG levels between 500 and 700 mg/dL in patients with recurrent infections and malignant blood disease?							
Clinical hematologists, n = 17	0.0	11.8	29.4	58.8	11.8	88.2	3.47
Clinical immunologists, n = 18	0.0	0.0	50.0	50.0	0.0	100.0	3.50
Overall, n = 35	0.0	5.7	40.0	54.3	5.7	94.3	3.49
Do you consider that trough IgG levels should be							

monitored to determine the correct dose of IVIG?							
Clinical hematologists, n = 17	0	29.4	23.5	47.1	29.4	70.6	3.18
Clinical immunologists, n = 18	0	0	5.6	94.4	0	100.0	3.94
Overall, n = 35	0	14.3	14.3	71.4	14.3	85.7	3.57
• Do you consider that early decision on IVIG replacement therapy is important to prevent the development or progression of bronchiectasis?							
Clinical hematologists, n = 17	5.9	11.8	41.2	41.2	17.7	82.4	3.18
Clinical immunologists, n = 18	0.0	0.0	33.3	66.7	0.0	100.0	3.67
Overall, n = 35	2.9	5.7	37.1	54.3	8.6	91.4	3.43

Table S8. Recommendation for the follow-up and monitoring of patients receiving intravenous IgG (IVIG) therapy

Questions	4-point Likert scale, % of responses				Consensus		Mean
	1 = Not necessary	2 = Optional	3 = Recommendable	4 = Indispensable	Against	In favor	
• During follow-up and monitoring of the patient treated with IVIG, do you consider that the following should be performed:							
Monitoring of IgG levels							
Clinical hematologists, n = 17	0	5.9	20.4	64.7	5.9	94.1	3.59
Clinical immunologists, n = 18	0	0	5.6	94.4	0	100.0	3.94
Overall, n = 35	0	2.9	17.1	80.0	2.9	97.2	3.77
Monitoring of clinical efficacy of IVIG (in terms of decreasing or preventing bacterial and viral infections)							
Clinical hematologists, n = 17	0.0	5.9	11.8	82.4	5.9	94.1	3.76
Clinical immunologists, n = 18	0.0	0.0	5.6	94.4	0.0	100.0	3.94
Overall, n = 35	0.0	2.9	8.6	88.6	2.9	97.1	3.86
• Frequency of monitoring of IgG levels							
Every 3 months							
Clinical hematologists, n = 17	0	17.7	58.8	23.5	17.6	82.3	3.06
Clinical immunologists, n = 18	5.6	11.1	50.0	33.3	16.7	83.3	3.11
Overall, n = 35	2.9	14.3	54.3	28.6	17.1	82.7	3.09
Every 6 months							
Clinical hematologists, n = 17	0	29.4	23.5	47.1	29.4	70.6	3.18
Clinical immunologists, n = 18	0	16.7	27.8	55.6	16.7	83.3	3.29
Overall, n = 35	0	22,9	25.7	51.4	22.9	77.1	3.29
Every 12 months							
Clinical hematologists, n = 17	23.5	41.2	11.8	23.5	64.7	35.3	2.35
Clinical immunologists, n = 18	22.2	27.8	22.2	27.8	50.0	50.0	2.56
Overall, n = 35	22.9	34.3	17.1	25.7	57.1	42.9	2.46
• Frequency of monitoring of the clinical efficacy of IVIG							
Every 3 months							
Clinical hematologists, n = 17	0	11.8	41.2	46.1	11.8	88.2	3.35
Clinical immunologists, n = 18	5.6	11.1	50.0	33.3	16.7	83.3	3.11
Overall, n = 35	2.9	11.4	45.7	40.0	14.3	85.7	3.23
Every 6 months							
Clinical hematologists, n = 17	0	17.7	35.3	47.1	17.7	82.4	3.29
Clinical immunologists, n = 18	0	16.7	27.8	55.6	16.7	83.3	3.39
Overall, n = 35	0	17.1	31.4	51.4	17.1	82.9	3.34
Every 12 months							
Clinical hematologists, n = 17	17.6	20.4	29.4	23.5	47.1	52.9	2.59
Clinical immunologists, n = 18	22.2	27.8	22.2	27.8	50.0	50.0	2.56
Overall, n = 35	20.0	28.6	25.7	25.7	48.6	5.4	2.57
• Treatment with IVIG should be discontinued in the following situations:							
After the end of chemotherapy							
Clinical hematologists, n = 17	23.5	47.1	23.5	5.9	70.7	29.4	2.12
Clinical immunologists, n = 18	38.9	50.0	5.6	5.6	88.9	11.1	1.78
Overall, n = 35	31.4	48.6	14.3	5.7	80.0	20.0	1.94
After 6 months without improvement of frequency or severity of bacterial infections							
Clinical hematologists, n = 17	5.9	41.2	47.1	5.9	47.1	52.9	2.53
Clinical immunologists, n = 18	16.7	38.9	33.3	11.1	55.6	44.4	2.39
Overall, n = 35	11.4	40.0	40.0	8.6	51.4	48.6	2.46
After 12 months without improvement of frequency or severity of bacterial infections							
Clinical hematologists, n = 17	0	41.2	23.5	35.3	41.2	58.8	2.94
Clinical immunologists, n = 18	22.2	38.9	22.2	16.7	61.1	38.9	2.33
Overall, n = 35	11.4	40.0	22.9	25.7	51.4	48.6	2.63
After recovery of IgG levels							
Clinical hematologists, n = 17	0	17.6	23.5	58.8	17.6	82.3	3.41
Clinical immunologists, n = 18	5.6	22.2	50.0	22.2	27.8	72.2	2.89
Overall, n = 35	2.9	20.0	37.1	40.0	22.9	77.1	3.14
